# Immunogenicity of SARS-CoV-2 spike antigens derived from Beta & Delta variants of concern

**DOI:** 10.1038/s41541-022-00540-7

**Published:** 2022-10-12

**Authors:** Bassel Akache, Tyler M. Renner, Matthew Stuible, Nazanin Rohani, Yuneivy Cepero-Donates, Lise Deschatelets, Renu Dudani, Blair A. Harrison, Christian Gervais, Jennifer J. Hill, Usha D. Hemraz, Edmond Lam, Sophie Régnier, Anne E. G. Lenferink, Yves Durocher, Michael J. McCluskie

**Affiliations:** 1 National Research Council Canada, Human Health Therapeutics, Ottawa, ON K1A 0R6 Canada; 2 National Research Council Canada, Human Health Therapeutics, Montreal, QC H4P 2R2 Canada; 3 National Research Council Canada, Aquatic and Crop Resource Development, Montreal, QC H4P 2R2 Canada

**Keywords:** Protein vaccines, Viral infection

## Abstract

Using our strongly immunogenic SmT1 SARS-CoV-2 spike antigen platform, we developed antigens based on the Beta & Delta variants of concern (VOC). These antigens elicited higher neutralizing antibody activity to the corresponding variant than comparable vaccine formulations based on the original reference strain, while a multivalent vaccine generated cross-neutralizing activity in all three variants. This suggests that while current vaccines may be effective at reducing severe disease to existing VOC, variant-specific antigens, whether in a mono- or multivalent vaccine, may be required to induce optimal immune responses and reduce infection against arising variants.

## Introduction

As the COVID-19 pandemic evolves with the continued emergence of new variants of concerns (VOC), the strategy to combat them with existing or new vaccines may need to be adapted. Through mutations in the spike protein sequence, certain VOCs have become more resistant to antibodies developed against the spike protein of the reference strain, originally identified in Wuhan^1–3^. As currently approved vaccines are based on the reference spike antigen, their efficacy against certain VOCs may be reduced^4,5^. Different vaccine-based approaches to combat these VOCs include (1) increasing antigen-specific antibody levels through additional boosts with reference strain-based vaccines to generate sufficient cross-neutralizing activity, (2) inducing T-cell responses to spike or other SARS-CoV-2 antigens (e.g., nucleocapsid and membrane proteins) and/or (3) utilizing spike-based antigens based on the VOC sequence to generate more efficient neutralizing activity against the targeted VOC^6–8^. While it may seem likely that the latter approach would generate better neutralization activity against a VOC, mutational differences in antigen sequence and/or quaternary structure could potentially reduce immunogenicity against critical spike regions, such as the receptor-binding domain.

We have previously demonstrated the immunogenicity and efficacy in preclinical models of vaccines containing SmT1 spike antigen, which consisted of a stabilized (pre-fusion containing dual proline mutations and abolished furin site) resistin-trimerized spike glycoprotein with His/FLAG affinity tags, produced in our CHO^2353™^ stable pool rapid production platform^9,10^. When based on the sequence of the Wuhan reference strain, adjuvanted SmT1 formulations induced similarly high neutralization activity against either reference or Alpha (B.1.1.7) variant spikes, but reduced neutralization against Beta (B.1.351) spike. Since affinity-tagged proteins would not be suitable for clinical use, we have now developed a next-generation tagless SmT1 antigen (SmT1v3) that can be purified efficiently using conventional chromatography resins. In the current study, we used our rapid, stable CHO pool production platform to produce tagless reference strain, as well as tagless Beta and Delta versions of the spike trimer, and evaluated them as vaccine antigens in a preclinical mouse model.

## Results

### Immunogenicity

**Fig. 1:**
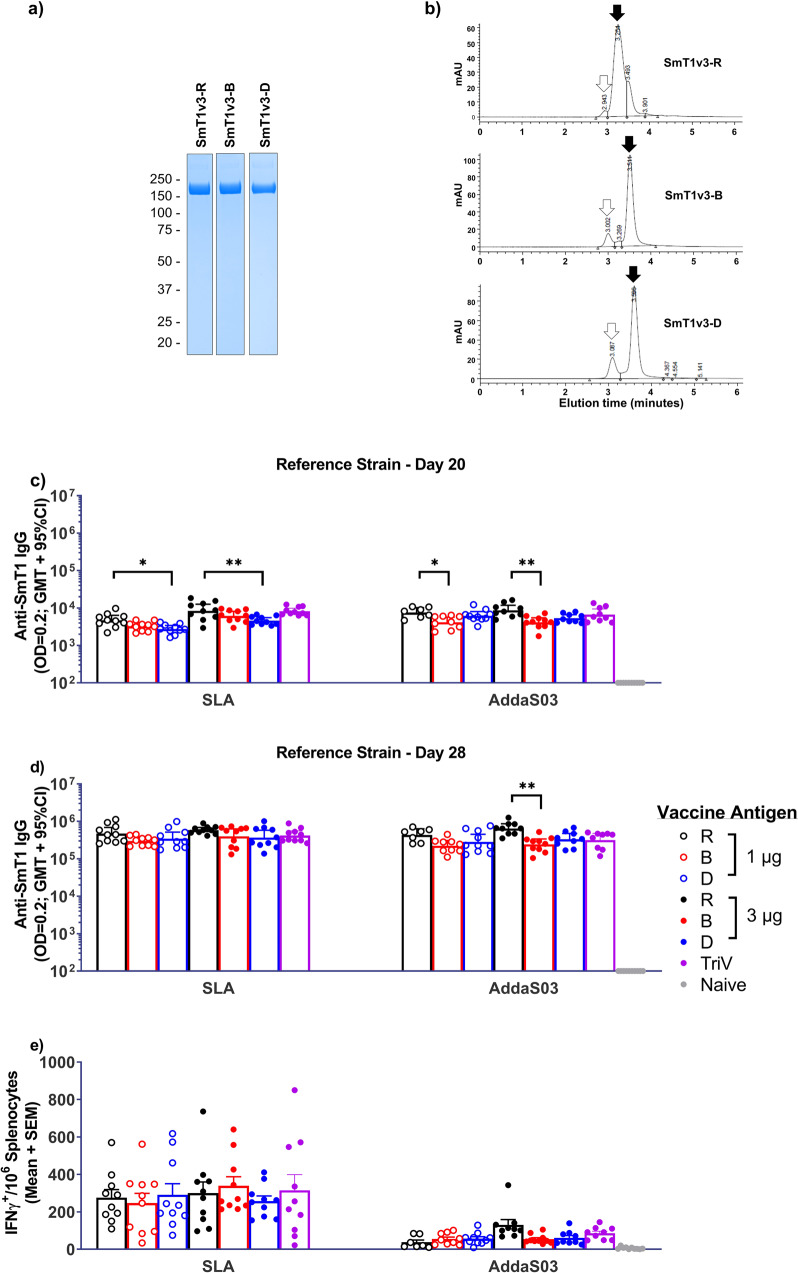
Humoral & cellular immune responses induced by VOC-based subunit vaccine formulations. SDS-PAGE (reducing conditions, 3 µg total protein, Coomassie stain, **a**) and UPLC-SEC profiles (**b**) of SmT1v3 reference strain ‘R’, Beta ‘B’ and Delta ‘D’ generated for vaccine formulations. Black and white arrows indicate trimer and hexamer protein populations, respectively, based on molecular weight estimates by MALS. C57BL/6 mice (*n* = 10/group) were immunized i.m. with SmT1v3 (R, B, and/or D) adjuvanted with SLA or AddaS03 on days 0 and 21. Serum was collected and analyzed by ELISA against tagged SmT1-R to determine the antibody titers on days 20 and 28 (**c**, **d**, respectively). Grouped data are presented as geometric mean + 95% confidence interval. Splenocytes were harvested on day 28 and analyzed by IFN-γ ELISpot when stimulated by spike peptide pools (**e**). Values obtained with media alone were subtracted from those measured in the presence of the peptides. Grouped data is presented as mean + standard error of mean (SEM). Statistical significance of differences for groups receiving formulations with the same adjuvant vs. the equivalent dose of SmT1v3-R is shown: *p < 0.05 & **p < 0.01 by one-way ANOVA followed by Tukey’s multiple comparisons test.

We generated untagged versions of SmT1 corresponding to (1) the reference strain: SmT1v3-R, (2) Beta VOC: SmT1v3-B, and (3) Delta VOC: SmT1v3-D. For all three constructs, final yields after purification were ~100–200 mg per liter of cell supernatant and spike protein recovery for the purification process was ~20–40% with >98% purity. Although the purity and molecular weight of the three spike constructs were very similar by sodium dodecyl sulfate–polyacrylamide gel electrophoresis (SDS-PAGE), their ultra-high performance liquid chromatography-size-exclusion chromatography (UPLC-SEC) profiles are distinct (Fig. 1a, b). As indicated by multi-angle light scattering (MALS) analysis of UPLC-SEC eluates, all three antigen types contain primarily trimeric spike protein (≥80%), but more hexamers were present in SmT1v3-B and -D protein preparations than in SmT1v3-R: SmT1v3-R (96% trimer; 3% hexamer), SmT1v3-B (88% trimer; 12% hexamer) and SmT1v3-D (80% trimer; 15% hexamer). The hexameric species, as characterized by molecular weight, are likely dimers of trimers, formed by specific inter-trimer interactions, as reported previously^11^. Notably, the shape of the trimer peak was also variant-dependent (for the SmT1v3 -B and -D protein preparations, the trimer peaks are sharper and slower-eluting). The relative levels of trimers vs. hexamers, as well as the characteristic UPLC-SEC profiles, were observed consistently for different production runs or when different purifications methods were used (data not shown). Together, these results indicate that intrinsic, variant-dependent differences exist in the structural conformations of the different spike proteins.

To assess the immunogenicity of these proteins, mice (*n* = 10 per group) were immunized at days 0 and 21 with vaccine formulations containing 1 or 3 µg of each of the various SmT1v3 constructs or with an admixed trivalent formulation (TriV) (1 µg of each of the three SmT1v3 constructs). To ensure that observed differences/trends were not adjuvant-specific, antigens were co-administered with either AddaS03, a mimetic of the squalene oil-in-water emulsion AS03, or sulfated lactosyl archaeol (SLA) archaeosomes, an experimental liposomal adjuvant composed of glycolipids containing archaeol and a sulfated lactose polar head group^12,13^. The dose levels and vaccine regimens were based on results from our previous study with SmT1^10^.

To confirm the immunogenicity of these various formulations, antigen-specific antibody and T cell levels were measured by ELISA and IFN-γ ELISpot, respectively (Fig. 1c–e). While some statistically significant differences were observed, the antibody responses to a reference-based spike protein were generally similar in groups receiving the same dose (i.e., 1 or 3 µg) of the various antigen constructs at day 20 (Fig. 1c). With the SLA-adjuvanted mono- and multivalent formulations, geometric mean titers (GMT) of 2856–5069 and 4739–8454 were detected with 1 and 3 µg of total antigen, respectively. Meanwhile, GMT of 4460–7917 and 4411–9328 were detected with AddaS03-adjuvanted 1 and 3 µg of total antigen, respectively. No significant differences in antibody titers were observed between SLA and AddaS03 when adjuvanting equivalent protein formulations. Following a second vaccine dose on day 21, antibody responses at day 28 did increase >1 log, but trends between the groups were similar to those observed at day 20 (Fig. 1d). Interestingly, similar trends were observed when ELISAs were conducted with Beta- or Delta- based spike protein (Supplementary Fig. 1). This is not surprising, as there are only small number of mutations in Beta or Delta Spike, with the study antigens sharing >98% identity in amino acid sequence. These assays measure total immune responses that cover the length of the spike antigen including the trimerization domain, and do not focus on the receptor-binding mutations known to alter the susceptibility of these VOCs to neutralizing antibodies. We have previously reported that vaccination with 1 and 3 µg of SmT1-based antigen provides similarity in responses by antibody ELISA, but not neutralization activity^10^. The number of IFN-γ^+^ splenocytes was similar regardless of antigen dose/construct with no statistically significant differences per particular adjuvant, but were markedly higher with SLA vs. AddaS03: mean number of IFN-γ^+^ SFCs/10^6^ splenocytes of 247–339 and 38–131 with SLA and AddaSO3, respectively (Fig. 1e). When comparing equivalent protein formulations, SLA induced significantly higher ELISpot responses than AddaS03 (*p* < 0.001) except in groups immunized with 3 µg of Reference SmT1 or the Trivalent formulation.

### Neutralization activity

**Fig. 2:**
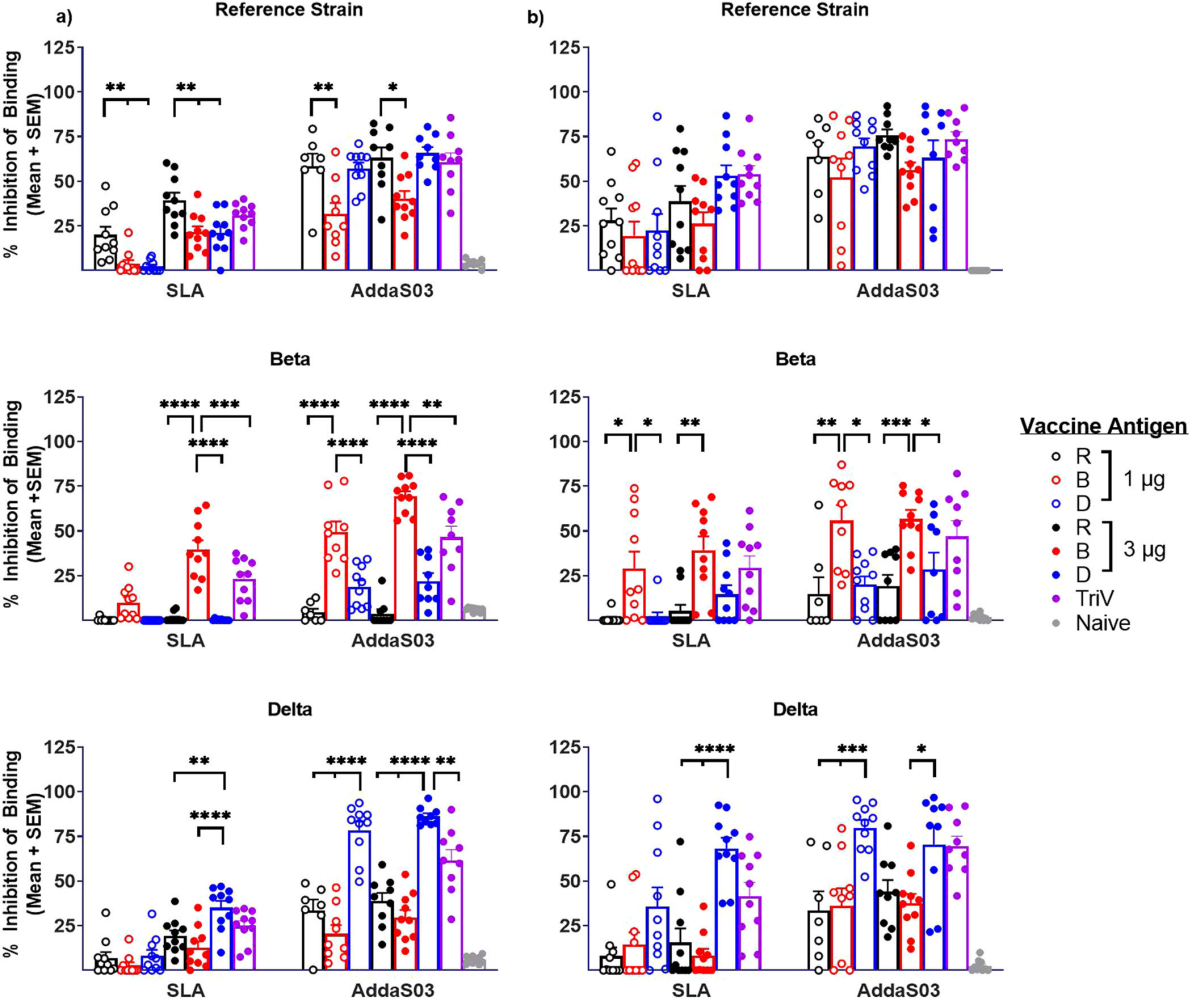
Neutralization activity induced by VOC-based subunit vaccine formulations. C57BL/6 mice (*n* = 10/group) were immunized i.m. with SmT1v3 reference strain ‘R’, Beta ‘B’ and/or Delta ‘D’ adjuvanted with SLA or AddaS03 on days 0 and 21. Serum from day 20 (**a**) at a final serum dilution of 1:25 and day 28 (**b**) at a final serum dilution of 1:250 were analyzed by SARS-CoV-2 Spike-ACE2 binding assay against the spike from the reference strain, Beta VOC or Delta VOC. Grouped data is presented as mean + standard error of mean (SEM). For statistical analysis, the significant differences are indicated between all groups receiving an equivalent dose of total antigen when compared against the group that received the antigen corresponding to the test strain: *p < 0.05, **p < 0.01, ***p < 0.001 and ****p < 0.0001 by one-way ANOVA followed by Tukey’s multiple comparisons test.

Neutralizing activity of immunized mouse sera from days 20 and 28 was next measured using a surrogate cell-based SARS-CoV-2 Spike-ACE2 binding assay previously shown to have a strong correlation to responses obtained with viral-based SARS-CoV-2 assays, namely plaque reduction neutralization test and in vivo hamster challenge model^10,14^. To allow for comparison to other publicly available sets of data, the neutralization activity reported as % inhibition of binding in Fig. 2 was also measured in IU/mL based on the World Health Organization (WHO) international standard (Supplementary Fig. 2). Of note, this standard has much higher neutralizing activity for an equivalent IU/mL against the reference strain of SARS-CoV-2 than the Beta and Delta variants. As such, an inflation of the IU/mL value against more resistant variants, such as Beta, was observed with the test samples.

Overall, stronger neutralization activity against a particular variant was observed when vaccinating with the corresponding antigen regardless of the adjuvant used with one exception: sera collected on day 28 in animals immunized with SmT1v3-B or SmT1v3-D generated statistically similar levels of neutralization as those immunized with SmT1v3-R (Fig. 2). At day 20, the trivalent formulation was able to induce neutralization to all three tested variants that were generally similar or better than that obtained with 1 µg of each antigen alone, but inferior to the activity obtained with 3 µg of SmT1v3-B or SmT1v3-D against Beta and Delta, respectively (Fig. 2a). For example, immunization with 1 or 3 µg of SmT1v3-B adjuvanted with SLA induced an average 10% (1429 international units (IU)/mL) and 40% (7013 IU/mL) inhibition against Beta-spike, respectively. TriV-SLA induced 23% inhibition (3541 IU/mL), while <5% inhibition (<300 IU/mL) against Beta-spike was measured following immunization with either 1–3 µg of SmT1v3-R or –D adjuvanted with SLA. In addition, inhibition against Delta spike was ≥79% (5226 IU/mL) for serum of mice immunized with either 1 or 3 µg of AddaS03-adjuvanted SmT1v3-D. The equivalent SmT1v3-R or –B-based formulations induced significantly lower inhibition activity of <40% (<1600 IU/mL) (p < 0.0001), while 62% (3248 IU/mL) was observed with TriV-AddaS03. Interestingly, some differences were also observed in the ability of the various antigen constructs to cross-neutralize: stronger neutralization against the Beta-spike was observed with 3 µg SmT1v3-D vs. SmT1v3-R when adjuvanted with AddaS03 (22 vs. 4%; *p* < 0.05 (3396 vs. 627 IU/mL)).

Neutralization activity following a second immunization was also measured using day 28 serum (Fig. 2b). Based on the higher antibody titers observed in Fig. 1d above, a lower concentration of day 28 serum was tested (1/250 vs. 1/25 at day 20), but it still resulted in similar or higher % inhibition of binding than observed in the same mice at day 20. Interestingly, while most of the trends were similar (i.e., neutralization activity against Beta or Delta spike was superior when immunizing with the corresponding SmT1v3-based antigen), some differences were observed when compared to the neutralization activity on day 20. For example, the activity following immunization with the trivalent formulation was no longer significantly lower than with the 3 µg single antigen formulations. In addition, no statistically significant differences were in seen in the activity against the reference strain. To further confirm the suitability of this assay to predict viral neutralization activity in our study, we analyzed a subset of samples across the study groups (representing an equal number dosed with the different antigens and/or adjuvants) in a SARS-CoV-2 spike-pseudotyped lentiviral-based neutralization assay to determine their levels of 50% neutralizing titer (NT50). A moderately strong correlation with an overall *R*^2^ = 0.5901 (*p* < 0.0001) across the three viral variants was observed between the NT_50_s and % inhibition of binding measured above (Supplementary Figure 3). This was markedly higher than the overall correlation between antibody titers and % inhibition of binding: *R*^2^ = 0.0171 (*p* = 0.2189). This indicates that cell-based SARS-CoV-2 Spike-ACE2 binding assay is a better predictor of the viral neutralization activity than antibody ELISA, probably due to the fact that non-neutralizing antibodies are contributing the majority of the overall signal in ELISA. There was an equally weak correlation between the NT_50_s and antibody titers *R*^2^ of 0.0001 (*p* = 0.8114; data not shown).

## Discussion

Several vaccines targeting SARS-CoV-2 have been shown to be efficacious in human clinical trials^15–18^. While all currently approved vaccines are based on the reference viral strain originally identified in Wuhan, they have also been shown to protect against disease from multiple VOC^4,5^ However, the ability of antibodies generated in vaccinated individuals to neutralize certain VOCs, namely Beta and Delta, is markedly reduced^1,2^. Most recently, Omicron and its sublineages have been shown to be even more resistant to immunity arising from SARS-CoV-2 vaccines^19^.

While immunizing with a VOC-based vaccine could theoretically enhance vaccine immunogenicity/efficacy against a particular VOC, this has not been previously demonstrated for SARS-CoV-2 subunit vaccines in a preclinical or clinical setting. Herein, we show that adjuvanted subunit vaccine formulations based on stable trimerized spike protein from the Beta and Delta VOCs were strongly immunogenic and able to induce stronger neutralization activity to the corresponding spike proteins. A recent report conducted with RNA-lipid nanoparticle vaccines, namely Moderna’s mRNA-1273, based on the spike protein from the reference and Beta strains has shown similar results^8^. They showed a higher level of Beta-specific neutralization in mice receiving a high dose of Beta-based vaccine. Interestingly, another mRNA vaccine study showed improved neutralization against Reference or Delta when immunized with the corresponding spike sequence, but no significant improvement with Beta mRNA^20^. Both groups illustrated that all vaccine types were able to protect mice from challenge with Reference, Beta, and Delta VOC. This data suggests that preclinical challenge models may not be sensitive enough to differentiate between VOC-based vaccines, probably due to the ability of high antibody titers and/or T cell responses to cross-neutralize different variants of SARS-CoV-2 in vivo. Due to the current predominance of the Omicron VOC (and its various sublineages), future studies will need to address the immunogenicity of Omicron-based antigens in monovalent vs. multivalent formulations. In addition, with the rapidly evolving nature of SARS-CoV-2, it is highly likely that new VOC will need to be targeted in the future. While the trends in neutralization activity between the SmT1-based antigens were similar when tested with two different adjuvants (SLA and AddaS03), some differences in the activities of the adjuvants were observed in our models with SLA and AddaS03 inducing higher T cell and neutralization activity, respectively. This could be due to differences in their mechanisms of action and is consistent with previous studies comparing the activity of SLA to a panel of adjuvants including oil-in-water type formulations^12^.

As the SARS-CoV-2 pandemic continues, we will be better able to assess the ability of reference strain-based vaccines to control existing and future VOCs. We and others have previously shown that repeated immunization with reference strain-based vaccine formulations can induce neutralizing activity to VOCs^6,10^. In addition, initial data indicates that subjects receiving a third dose of a reference strain-based vaccine may be better protected from infection by VOCs such as Delta^21^. Our results suggest that moving towards antigens based on the specific sequence of a VOC could enhance the specific immunogenicity towards that particular variant and potentially maximize vaccine efficacy, especially against highly resistant VOCs. Multivalent formulations could simultaneously provide wider protection against multiple VOCs and may offer better protection against new future variants, which will likely share particular mutations present in current VOCs. It will be important to assess these approaches in a clinical setting, in both naïve and pre-vaccinated individuals, as a high percentage of the population in certain parts of the world already has some level of immunogenicity against SARS-CoV-2. With the persistent emergence of new variants, the ability to produce newly designed antigens rapidly will be critical. We believe that our production platform, which could generate high levels of spike vaccine antigen appropriate for clinical trials in <6 weeks by using GMP-banked CHO cells to prepare stable pools, is ideal in this regard.

## Materials and methods

### Antigens

SmT1v3-R, -B, and -D constructs are based on SARS-CoV-2 spike trimers described previously^22,23^, but with C-terminal FLAG/His affinity tags removed. Briefly, the SARS-CoV-2 reference strain spike ectodomain sequence (amino acids 1-1208 derived from Genbank accession number MN908947) was codon-optimized for Chinese Hamster Ovary (CHO) cells and synthesized by GenScript. Within the construct, the spike glycoprotein was preceded by its natural N-terminal signal peptide and fused at the C-terminus to human resistin (accession number NP_001180303.1, amino acids 23-108). Mutations were added to stabilize the generated spike protein as previously described; amino acids 682-685 (RRAR) and 986-987 (KV) were replaced with GGAS and PP, respectively^24,25^. Constructs were then cloned into the pTT241 plasmid. Expression constructs for VOC spike variants were prepared by re-synthesizing and replacing restriction fragments encompassing mutations present in the Beta (SmT1v3-B) (D80A, D215G, 241del, 242del, 243del, K417N, E484K, N501Y, D614G, A701V) and Delta (SmT1v3-D) (T19R, G142D, E156-, F157-, R158G, L452R, T478K, D614G, P681R, D950N) variants, while maintaining the codon-optimized sequences of the remaining amino acids used for SmT1v3-R expression. Stably transfected pools were established by MSX selection using the CHO^2353™^ cell line and used for 10-day fed-batch productions with cumate induction as described^23^. Spike proteins were purified using a proprietary multi-step non-affinity-based process and formulated in Dulbecco’s Phosphate-Buffered Saline (DPBS; Hyclone, Logan, Utah, USA) adjusted to pH 7.8 at protein concentrations of 1.1–1.4 mg/ml. Purified proteins were analyzed by SDS-PAGE and analytical SEC-UPLC. SEC-UPLC was run on an Acquity H-Class Bio UPLC system (Wyatt Technology, Santa Barbara, CA, USA) in phosphate-buffered saline (PBS) + 0.02% Tween-20 on a 4.6 × 300 mm Acquity BEH450 column (2.5 μm bead size; Waters Limited, Mississauga, ON, Canada) coupled to a miniDAWN MALS detector and Optilab T-rEX refractometer (Wyatt). The identity and purity of the antigens were also confirmed by mass spectrometry. The absence of endotoxin contamination was verified using Endosafe cartridge-based Limulus amebocyte lysate tests (Charles River Laboratories, Charleston, SC, USA). The unprocessed gel images are available in the Source Data file deposited on the Dryad Data Repository (10.5061/dryad.qjq2bvqk9).

### Institutional review board statement

Mice were maintained at the small animal facility of the National Research Council (NRC) Canada in accordance with the guidelines of the Canadian Council on Animal Care. All procedures performed on animals in this study were approved by our Institutional Review Board (NRC Human Health Therapeutics Animal Care Committee) and covered under animal use protocol 2020.10. All experiments were carried out in accordance with the ARRIVE guidelines.

### Immunization and sample collection

Female C57BL/6 mice (6–8 weeks old) were obtained from Charles River Laboratories (Saint-Constant, Canada). Animals were maintained at the small animal facility of the NRC Canada in accordance with the guidelines of the Canadian Council on Animal Care.

Mouse experiments (*n* = 10 per group) consisted of two separate equal-sized cohorts, where animals were treated identically but had procedures conducted on different days. Data from both cohorts were combined and included for analysis. Seven out of a total of 70 mice receiving AddaS03-adjuvanted formulations had to be excluded due to high local reactogenicity at the site of injection. As such, the AddaS03 groups had 9–10 mice per group included in the final analysis, except for one group (1 µg SmT1v3-R) where results from 7 mice were included. Antigen and adjuvant vaccine components were admixed and diluted in PBS (Thermo Fisher Scientific, Waltham, MA, USA) prior to administration in a final volume of 50 µL per dose. SLA archaeosomes are proprietary NRC adjuvants that were prepared as previously described^26^. Levels of endotoxin in the SLA archaeosomes were verified by the Endosafe® cartridge-based Limulus amebocyte lysate test (Charles River Laboratories) and confirmed to be <0.1 EU per mg. AddaS03 (Invivogen, San Diego, CA, USA) was prepared as per the manufacturer’s instructions.

Animals were immunized by intramuscular (i.m.) injection (50 µL) into the left tibialis anterior muscle on days 0 and 21 with various vaccine formulations as described above. On day 28, mice were anesthetized with isoflurane and then euthanized by cervical dislocation prior to collection of spleens for measurement of cellular immune responses by IFN-γ ELISpot. Mice were bled via the submandibular vein on days 20 and 28 with recovered serum used for quantification of antigen-specific IgG antibody levels and neutralization assays. Samples were simultaneously collected from 10 naïve animals for the assessment of background immune responses. Each of the samples from the individual mice was tested separately in the various readouts.

### Anti-spike IgG ELISA

Anti-spike total IgG titers in serum were measured by indirect ELISA with SmT1-R, -B, or -D as previously described^10^. Briefly, 96–well high-binding ELISA plates (Thermo Fisher Scientific) were coated with 0.3 µg/mL SmT1 protein diluted in PBS. Serum samples were serially diluted 3.162-fold and added to the plates to allow for binding of antibodies to the protein. Bound IgG was detected with goat anti-mouse IgG -HRP (1:4000, Southern Biotech, Birmingham, AL, USA) prior to the addition of the substrate o-phenylenediamine dihydrochloride (Sigma-Aldrich). Bound IgG Abs were detected spectrophotometrically at 450 nm. Titers for IgG in serum were defined as the dilution that resulted in an absorbance value (OD_450_) of 0.2 and was calculated using XLfit software (ID Business Solutions, Guildford, UK). Samples that did not reach the target OD were assigned the value of the lowest tested dilution (i.e., 100) for analysis purposes. No detectable titers were measured in serum samples from naïve control animals.

### IFN- γ ELISpot

IFN- γ ELISpot was also conducted as previously described^10^. The levels of spike glycoprotein specific T cells were quantified by ELISpot using a mouse IFN-γ kit (Mabtech Inc., Cincinnati, OH, USA). A spike peptide library (JPT Peptide Technologies GmbH) based on the reference strain sequence and consisting of 315 peptides (15mers overlapping by 11 amino acids with last peptide consisting of a 17mer) was used to stimulate splenocytes isolated from each of the mice. The library was split into three subpools and used to separately stimulate 4 × 10^5^ cells in duplicate at a final concentration of 2 µg/mL per peptide. Cells were also incubated without any stimulants to measure background responses. Spots were counted using an automated ELISpot plate reader (Cellular Technology LTD, Beachwood, OH, USA). For each animal, values obtained with media alone were subtracted from those obtained with each of the spike peptide pools, and then combined to yield an overall number of antigen-specific IFN-γ^+^ SFC/10^6^ splenocytes per animal.

### Cell-based SARS-CoV-2 Spike-ACE2 binding assay

The ability serum to neutralize the binding of labeled SARS-CoV-2 spike trimers (SmT1) to Vero E6 cells was measured as previously described^10^. Indicated dilutions of mouse serum were mixed with 250 ng of biotinylated spike and 1 × 10^5^ Vero E6 cells (ATCC® CRL-1586™). The amount of bound spike was quantified using a Streptavidin-phycoerythrin conjugate prior to acquisition on an LSR Fortessa (Becton Dickinson). For analysis purposes, samples with calculated values ≤0 were assigned a value of 0. The levels of neutralization were normalized to the World Health Organization human standard reference material (20/136 from NIBSC, South Mimms, UK) to obtain the anti-SARS-CoV-2 activity (IU/mL) in undiluted mouse serum.

### Pseudovirus neutralization assay

Pseudovirus neutralization assay was performed in 384-well plate format adapted from previously described protocol and modification^27,28^. Briefly, 4-fold serial dilutions of the serum samples were incubated with diluted virus at a 2:1 ratio for 1 h at 37 °C before addition to HEK293-ACE2/TMPRSS2 cells obtained from BEI Resources repository of ATCC and the NIH (NR-55293). Infectivity was then measured by luminescence readout per well. Bright-Glo luciferase reagent (Promega, E2620) was added to wells for 2 min before reading with a PerkinElmer Envision instrument. Neutralization Titer 50 (NT_50_) were calculated with nonlinear regression (log[inhibitor] versus normalized response – variable slope) with the 100% and 0% constraint. Pseudotyped lentiviral particles were produced expressing the SARS-CoV-2 variant spikes under CMV promotor and were packaged onto lentiviral vectors obtained through BEI Resources, NIAID, NIH: SARS-Related Coronavirus 2, Wuhan-Hu-1 (GenBank # NC_045512) Spike-Pseudotyped Lentiviral Kit, NR-52948. pcDNA3.3-SARS2-B.1.617.2 expressing the SARS-CoV-2 B.1.617.2 (Delta variant) (Addgene plasmid # 172320) and pcDNA3.3_CoV2_501V2 expressing SARS-CoV-2, B.1.351 (Beta variant) (Addgene plasmid # 170449) spike proteins were gifts from David Nemazee.

### Statistical analysis

Data were analyzed using GraphPad Prism® version 8 (GraphPad Software). Statistical significance of the difference between groups was calculated by one-way analysis of variance followed by post hoc analysis using Tukey’s (comparison across all groups) multiple comparison test. Data were log-transformed (except for data from Spike-ACE2 binding assay) prior to statistical analysis. For all analyses, differences were considered to be not significant with *p* > 0.05. Significance was indicated in the graphs as follows: *p < 0.05, **p < 0.01, ***p < 0.001, and ****p < 0.0001. To correlate data sets, *R*^2^ values were determined by fitting the points to a regression line and *p* values (two-tailed) were determined using a Spearman correlation.

### Reporting summary

Further information on research design is available in the Nature Research Reporting Summary linked to this article.

## Supplementary information

Supplementary Information

REPORTING SUMMARY

## Data Availability

The data and unprocessed gel images are available via the Dryad Data Repository (10.5061/dryad.qjq2bvqk9).
