# Supplementary material for: Immunogenicity of SARS-CoV-2 spike antigens derived from Beta & Delta variants of concern
Source: NPJ Vaccines. 2022 Oct 12;7:118. doi: 10.1038/s41541-022-00540-7 (PMC9555707; doi:10.1038/s41541-022-00540-7)
Supplement: Supplementary file 1 — Supplementary Information [file 41541_2022_540_MOESM1_ESM.pdf]

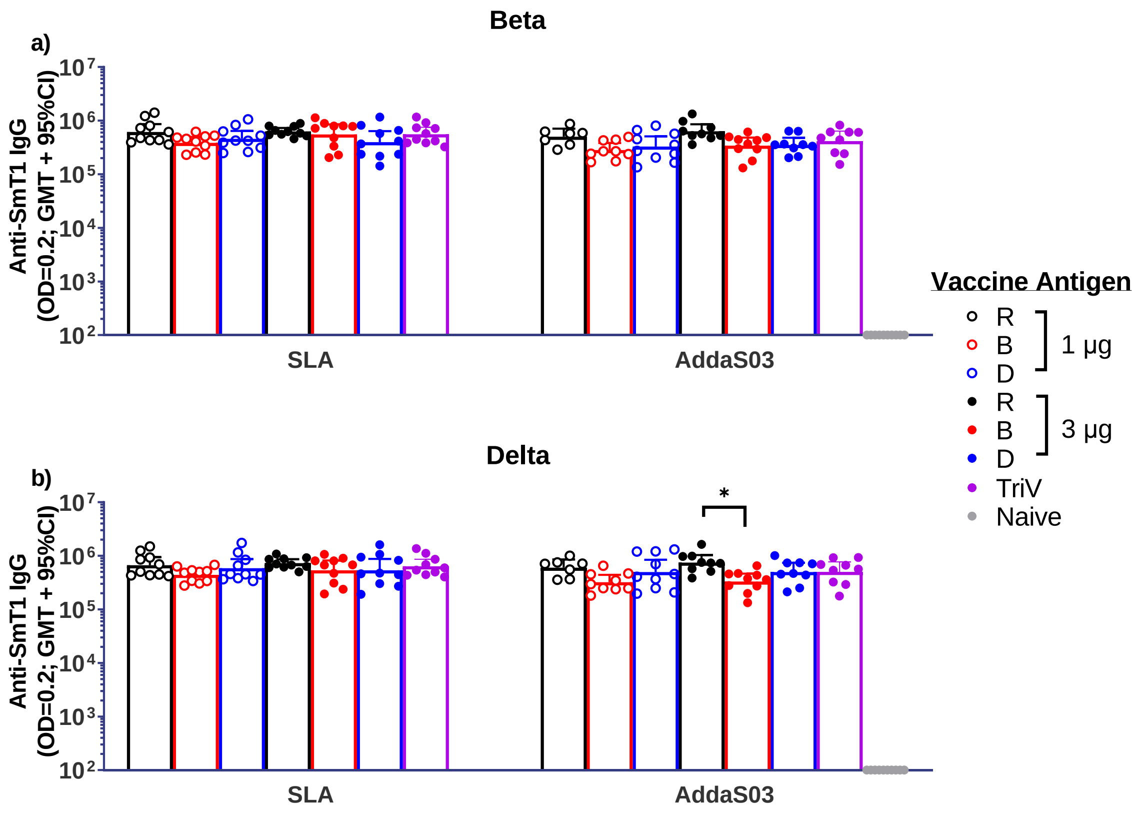

Supplementary Figure 1: **Humoral immune responses to Beta- and Delta-based Spike protein.**

C57BL/6 mice (n = 10/group) were immunized i.m. with SmT1v3 (R, B and/or D) adjuvanted with SLA or AddaS03 on Days 0 and 21. Serum was collected at Day 28 and analyzed by ELISA against tagged SmT1-B (Panel a) or SmT1-D (Panel b) to determine the antibody titers. Grouped data is presented as geometric mean + 95% confidence interval. Statistical significance of differences vs. the equivalent dose of SmT1v3-R is shown: *: $p<0.05$ by one-way ANOVA followed by Tukey's multiple comparisons test.

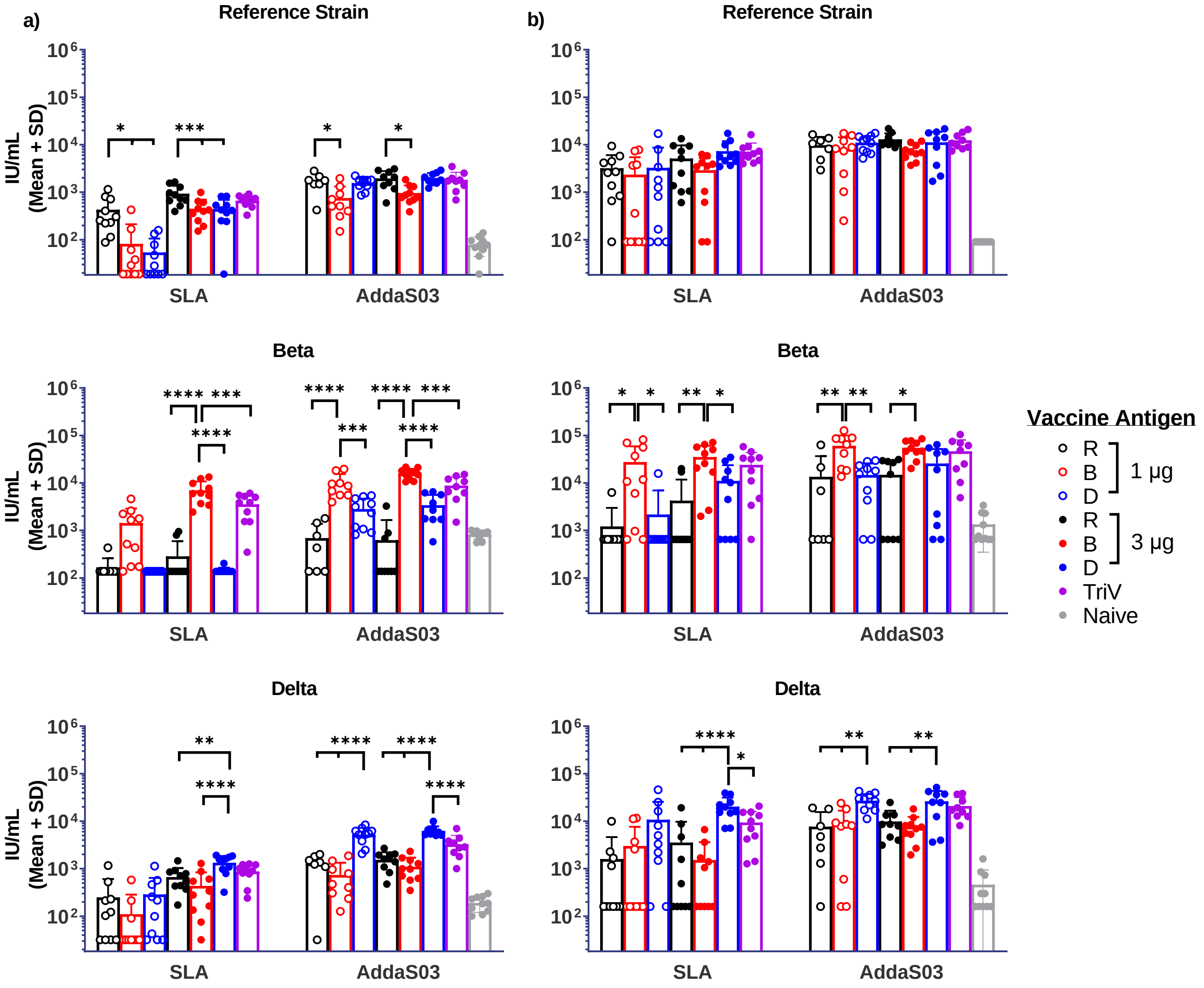

Supplementary Figure 2: Neutralization activity induced by VOC-based subunit vaccine formulations.

C57BL/6 mice (n = 10/group) were immunized i.m. with SmT1v3 reference strain 'R', Beta 'B' and/or Delta 'D' adjuvanted with SLA or AddaS03 on Days 0 and 21. Serum from Day 20 (Panel a) at a final serum dilution of 1:25 and Day 28 (Panel b) at a final serum dilution of 1:250 were analyzed Spike-ACE2 binding assay against the spike from the reference strain, Beta VOC or Delta VOC. The neutralization activity is calculated as international units per millilitre (IU/mL) as related to the WHO international standard (NIBSC 20/136). Grouped data is presented as mean + standard deviation (SD). For statistical analysis, the significant differences are indicated between all groups receiving an equivalent dose of total antigen when compared against the group that received the antigen corresponding to the test strain: *: $p<0.05$ & **: $p<0.01$, ***: $p<0.001$ and ****: $p<0.0001$ by one-way ANOVA followed by Tukey's multiple comparisons test.

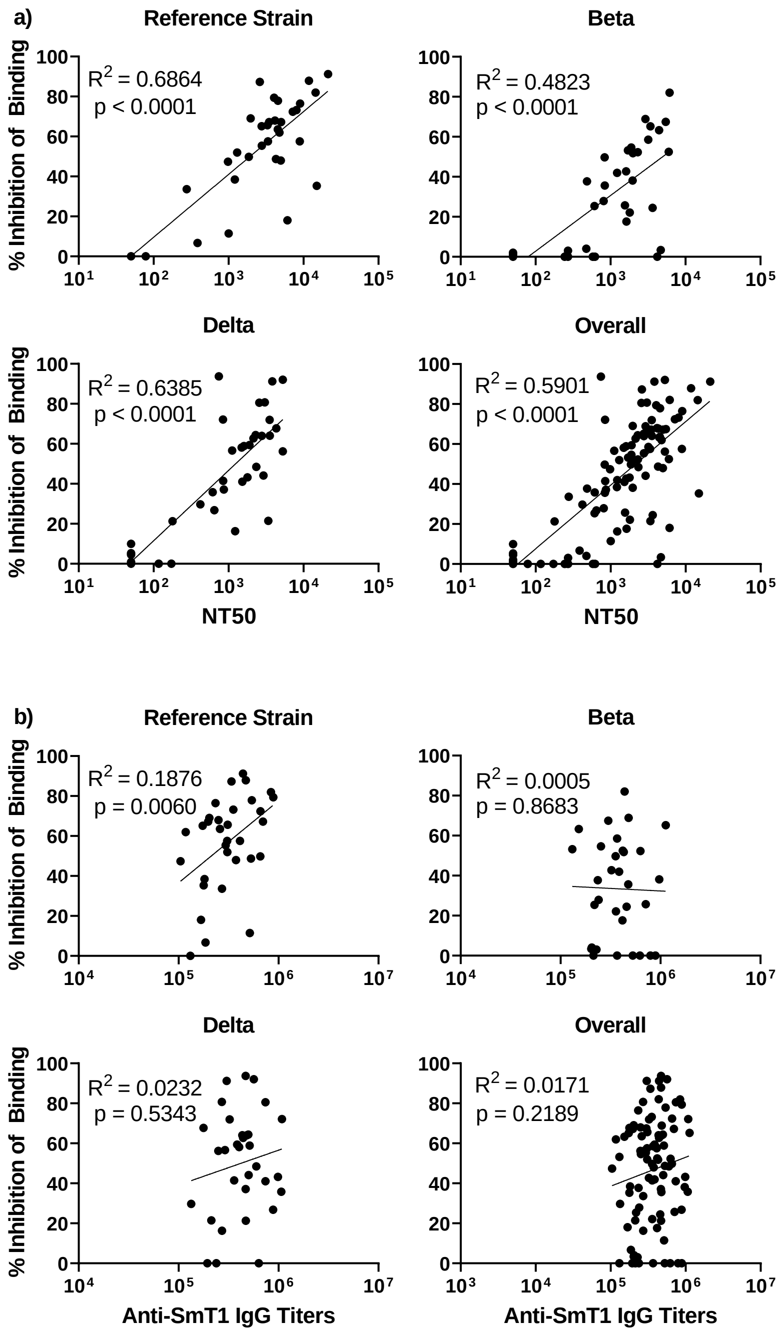

Supplementary Figure 3: Correlation of neutralization activity with SARS CoV-2 Spike-ACE2 binding assay to pseudovirus neutralization assay or IgG ELISA.

C57BL/6 mice were immunized i.m. with SmT1v3 reference strain 'R', Beta 'B' and/or Delta 'D' adjuvanted with SLA or AddaS03 on Days 0 and 21. The correlation between data obtained for a particular sample against matching spike protein (R, B or D) with the Spike-ACE2 binding assay and the NT_{50}s measured in the pseudovirus neutralization assay (Panel a) or anti-SmT1 IgG Titers measured by antibody ELISA (Panel b) from Day 28 serum was determined. The correlation is shown by variant or combined all together.
