# Supplementary material for: Immunogenicity of SARS-CoV-2 spike antigens derived from Beta & Delta variants of concern
Source: NPJ Vaccines. 2022 Oct 12;7:118. doi: 10.1038/s41541-022-00540-7 (PMC9555707; doi:10.1038/s41541-022-00540-7)
Supplement: Supplementary file 2 — REPORTING SUMMARY [file 41541_2022_540_MOESM2_ESM.pdf]

nature portfolio

Corresponding author(s): Michael J McCluskie

Last updated by author(s): Jul 1, 2022

Reporting Summary

Nature Portfolio wishes to improve the reproducibility of the work that we publish. This form provides structure for consistency and transparency in reporting. For further information on Nature Portfolio policies, see our Editorial Policies and the Editorial Policy Checklist.

Statistics

For all statistical analyses, confirm that the following items are present in the figure legend, table legend, main text, or Methods section.

n/a	Confirmed	
☐	☒	The exact sample size (*n*) for each experimental group/condition, given as a discrete number and unit of measurement
☐	☒	A statement on whether measurements were taken from distinct samples or whether the same sample was measured repeatedly
☐	☒	The statistical test(s) used AND whether they are one- or two-sided<br>*Only common tests should be described solely by name; describe more complex techniques in the Methods section.*
☐	☒	A description of all covariates tested
☐	☒	A description of any assumptions or corrections, such as tests of normality and adjustment for multiple comparisons
☐	☒	A full description of the statistical parameters including central tendency (e.g. means) or other basic estimates (e.g. regression coefficient) AND variation (e.g. standard deviation) or associated estimates of uncertainty (e.g. confidence intervals)
☐	☒	For null hypothesis testing, the test statistic (e.g. *F*, *t*, *r*) with confidence intervals, effect sizes, degrees of freedom and *P* value noted<br>*Give P values as exact values whenever suitable.*
☒	☐	For Bayesian analysis, information on the choice of priors and Markov chain Monte Carlo settings
☒	☐	For hierarchical and complex designs, identification of the appropriate level for tests and full reporting of outcomes
☐	☒	Estimates of effect sizes (e.g. Cohen's *d*, Pearson's *r*), indicating how they were calculated

Our web collection on statistics for biologists contains articles on many of the points above.

Software and code

Policy information about availability of computer code

Data collection	FACSDiva v9.0, CTL Immunospot 7.0.28.4, Softmax pro 6.3
Data analysis	FlowJo v10.8.1, Graphpad 9.4.0, XLFit5

For manuscripts utilizing custom algorithms or software that are central to the research but not yet described in published literature, software must be made available to editors and reviewers. We strongly encourage code deposition in a community repository (e.g. GitHub). See the Nature Portfolio guidelines for submitting code & software for further information.

Data

Policy information about availability of data

All manuscripts must include a data availability statement. This statement should provide the following information, where applicable:

- Accession codes, unique identifiers, or web links for publicly available datasets
- A description of any restrictions on data availability
- For clinical datasets or third party data, please ensure that the statement adheres to our policy

The data presented in this study are available on request from the corresponding author. The data are not publicly available due to privacy concerns.

March 2021

Human research participants

Policy information about studies involving human research participants and Sex and Gender in Research.

Reporting on sex and gender	Not applicable
Population characteristics	Not applicable
Recruitment	Not applicable
Ethics oversight	Not applicable

Note that full information on the approval of the study protocol must also be provided in the manuscript.

Field-specific reporting

Please select the one below that is the best fit for your research. If you are not sure, read the appropriate sections before making your selection.

☒ Life sciences ☐ Behavioural & social sciences ☐ Ecological, evolutionary & environmental sciences

For a reference copy of the document with all sections, see nature.com/documents/nr-reporting-summary-flat.pdf

Life sciences study design

All studies must disclose on these points even when the disclosure is negative.

Sample size	N=10/group of mice was selected based on our previous experience with this immunogenicity model. This would provide sufficient number of mice per group to enable robust statistical analysis
Data exclusions	Seven out of a total of 70 mice receiving AddaS03-adjuvanted formulations had to be excluded due to high local reactogenicity at the site of injection. As such, the AddaS03 groups had 9-10 mice per group included in the final analysis, except for one group (1 µg SmT1v3-R) where results from 7 mice were included.
Replication	Mouse experiments (n=10 per group) consisted of 2 separate equal sized cohorts, where animals were treated identically but had procedures conducted on different days. Data from both cohorts was combined and included for analysis.
Randomization	Mice were randomly assigned to various groups upon arrival. No factors were taken into consideration when deciding which group to assign them to.
Blinding	Investigators were blinded and not aware of nature of treatment per group when collecting/analyzing data.

Reporting for specific materials, systems and methods

We require information from authors about some types of materials, experimental systems and methods used in many studies. Here, indicate whether each material, system or method listed is relevant to your study. If you are not sure if a list item applies to your research, read the appropriate section before selecting a response.

Materials & experimental systems

n/a	Involved in the study	
☐	☒	Antibodies
☐	☒	Eukaryotic cell lines
☒	☐	Palaeontology and archaeology
☐	☒	Animals and other organisms
☒	☐	Clinical data
☒	☐	Dual use research of concern

Methods

n/a	Involved in the study	
☒	☐	ChIP-seq
☐	☒	Flow cytometry
☒	☐	MRI-based neuroimaging

Antibodies

Antibodies used	Anti-mouse IgG-Horseradish peroxidase (Southern Biotech Cat. no. 1030-05)

Validation	*Describe the validation of each primary antibody for the species and application, noting any validation statements on the manufacturer's website, relevant citations, antibody profiles in online databases, or data provided in the manuscript.*

Eukaryotic cell lines

Policy information about cell lines and Sex and Gender in Research

Cell line source(s)	Vero E6 VERO C1008 [Vero 76, clone E6, Vero E6] CRL-1586™ ATCC<br>HEK293-ACE2/TMPRSS2 cells BEI Resources repository of ATCC and the NIH (NR-55293)
Authentication	Morphology was authenticated via microscopy.
Mycoplasma contamination	We confirm that the cell lines were tested for mycoplasma via PCR and was negative.
Commonly misidentified lines<br>(See ICLAC register)	*Name any commonly misidentified cell lines used in the study and provide a rationale for their use.*

Animals and other research organisms

Policy information about studies involving animals; ARRIVE guidelines recommended for reporting animal research, and Sex and Gender in Research

Laboratory animals	C57Bl/6 female mice (6-8 weeks old) from Charles River
Wild animals	N/A
Reporting on sex	Findings do not apply to a particular sex. Immune responses to these types of vaccine formulations have been previously shown to be similar in male and female mouse models.
Field-collected samples	Not applicable
Ethics oversight	Animals were maintained at the small animal facility of the National Research Council Canada (NRC) in accordance with the guidelines of the Canadian Council on Animal Care. All procedures performed on animals in this study were approved by our Institutional Review Board (NRC Human Health Therapeutics Animal Care Committee) and covered under animal use protocol 2020.10. All experiments were carried out in accordance with the ARRIVE guidelines.

Note that full information on the approval of the study protocol must also be provided in the manuscript.

Flow Cytometry

Plots

Confirm that:

- [ ] The axis labels state the marker and fluorochrome used (e.g. CD4-FITC).
- [ ] The axis scales are clearly visible. Include numbers along axes only for bottom left plot of group (a 'group' is an analysis of identical markers).
- [ ] All plots are contour plots with outliers or pseudocolor plots.
- [ ] A numerical value for number of cells or percentage (with statistics) is provided.

Methodology

Sample preparation	Briefly, the ability of labeled SARS-CoV-2 spike trimers (SmT1) to bind the surface of Vero E6 cells following co-incubation with sera/plasma was measured. Vero E6 cells were maintained in RPMI 1640 supplemented with 10% FBS, 1% penicillin/ streptomycin, 20 mM HEPES, 1×non-essential amino acids, 1×Glutamax, 50 µM 2-mercaptoethanol (all from Thermo Fisher Scientific) at 37 °C with 5% CO2. Soluble SmT1, was biotinylated and isolated from free biotin using EZ-Link™ NHS-LC-LC-Biotin (Thermo Fisher Scientific) according to manufacturer's instructions. Indicated dilutions of mouse/hamster serum or convalescent human plasma (20/130, 20/162, 20/B764 from NIBSC, South Mimms, UK) were mixed with 250 ng of biotinylated spike and 1×105 Vero E6 cells (ATCC® CRL-1586™) in the presence of 0.05% azide within a 96-well V-bottom plate (Nunc™, Thermo Fisher Scientific) and incubated for 1 h at 4 °C, while protected from light. Regardless of serum concentration, the final volume of all samples was normalized to 150 µL. Cells were washed with PBS+1% bovine serum albumin (BSA)+0.05% Azide and incubated with Streptavidin–phycoerythrin conjugate for 1 h at 4 °C (Thermo Fisher Scientific). After another wash, the cells were fixed using CytoFix™ (Becton Dickinson, Franklin Lakes, NJ, USA) and resuspended in wash buffer+5 mM EDTA for acquisition on an LSR Fortessa (Becton Dickinson). Spike binding to cells was determined by calculating the Geometric Mean Fluorescence Intensity (MFI) of PE (on singlet cell population) and subtracting the same parameter measured from control cells (incubated in absence of plasma/serum), both above background noise as determined by the negative control, using FlowJo analysis software. Percent neutralization was calculated as follows: % neutralization=100 – (100 × (Geometric MFI for PE of test sample – Geometric MFI for PE of negative control sample (i.e. cells incubated only with Streptavidin-PE and without spike protein)/(Geometric MFI for PE of positive control sample (i.e., cells incubated with spike without serum/plasma) – Geometric MFI for PE of negative control sample)). For analysis purposes,

	samples with calculated values≤0 were assigned a value of 0.
Instrument	LSR II Fortessa SORP
Software	FACSDiva v9.0 and FlowJo v10.8.1
Cell population abundance	Typically at least 60% singlets of Vero E6 after 40um filtration.
Gating strategy	FSC / SSC gate was designed to highlight the majority of cells and negate aggregates and debris<br>FSC H / FSC A gate was designed to isolate singlet cells in a diagonal linear fashion<br>No other gating was used. MFI of the singlet population was used to determine binding of fluorophore.

☐ Tick this box to confirm that a figure exemplifying the gating strategy is provided in the Supplementary Information.
